# Nomogram for Acute Bilirubin Encephalopathy Risk in Newborns With Extreme Hyperbilirubinemia

**DOI:** 10.3389/fneur.2020.592254

**Published:** 2020-11-24

**Authors:** Yangming Qu, Shuhan Huang, Xin Fu, Youping Wang, Hui Wu

**Affiliations:** Department of Neonatology, The First Hospital of Jilin University, Changchun, China

**Keywords:** acute bilirubin encephalopathy, newborn, nomogram, risk factors, predict

## Abstract

**Background and Objectives:** This work aimed to develop a predictive model of neonatal acute bilirubin encephalopathy.

**Methods:** We retrospectively analyzed the data on extreme hyperbilirubinemia (EHB) newborns hospitalized in the First Hospital of Jilin University from January 1, 2012 to December 31, 2019. The demographic characteristics, pathological information, and admission examination results of newborns were collected to analyze the factors affecting acute bilirubin encephalopathy and to establish a predictive model.

**Results:** A total of 517 newborns were included in this study, of which 102 (19.7%) had acute bilirubin encephalopathy. T1WI hyperintensity [18.819 (8.838–40.069)], mother's age > 35 years [2.618 (1.096–6.2530)], abnormal white blood cell (WBC) [6.503 (0.226–18.994)], TSB level [1.340 (1.242–1.445)], and albumin level [0.812 (0.726–0.907)] were independently associated with neonatal acute bilirubin encephalopathy (ABE). All independently associated risk factors were used to form an ABE risk estimation nomogram. The bootstrap validation method was used to internally validate the resulting model. The nomogram demonstrated good accuracy in predicting the risk of ABE, with an unadjusted C index of 0.943 (95% CI, 0.919–0.962) and a bootstrap-corrected C index of 0.900.

**Conclusion:** A nomogram was constructed using five risk factors of ABE. This model can help clinicians determine the best treatment for neonatal hyperbilirubinemia.

## Introduction

Hyperbilirubinemia is a common disease in newborns, occurring in more than 84% of late preterm and term neonates (1). Although most patients had cases that resolved by 2 weeks of age, some developed severe complications. Neonatal acute bilirubin encephalopathy (ABE) is one of the most potentially devastating complications, usually resulting in permanent damage to the central nervous system (2). The incidence of ABE may have decreased in developed countries in recent years, but it still occurs at a rate of 0.4 to 2.7 cases per 100,000 infants (3, 4), with a higher incidence in Asia, the Middle East, and Africa (5). In theory, this is an entirely avoidable problem if we can predict and intervene early. Studies (6–8) have shown that ABE is associated with increased total serum bilirubin (TSB) concentration. The NIHCD proposes to define TSB ≥25mg/dl as very severe hyperbilirubinemia (EHB) because of the increased risk of bilirubin brain damage in these patients (9). The American Academy of Pediatrics (AAP) recommends that all newborns with gestation ≥35 weeks and TSB ≥25mg/dl be actively treated to reduce the risk of ABE (10). However, TSB is a poor indicator of specificity. Some newborns may not have ABE, but they received too much treatment when TSB was used as a predictor (11, 12). An expert panel recommended the use of TSB or transcutaneous bilirubin in combination with clinical risk factors as an indicator for discharge evaluation in neonates with hyperbilirubinemia (8). The most significant clinical risk factors have not yet been identified. Therefore, it is necessary to identify the related factors to develop a predictive model of ABE to make early prediction and timely intervention for the disease.

## Methods

### Study Design

We retrospectively analyzed the data on extreme hyperbilirubinemia newborns hospitalized in the First Hospital of Jilin University from January 1, 2012 to December 31, 2019. The inclusion criteria were as follows: TSB ≥ 25mg/dl (428 μmol/L), gestational age (GA) ≤ 35 weeks, and age at admission ≤14 days. The exclusion criteria were as follows: conjugated bilirubin >20% TSB, intracranial infection, hypoxic–ischemic encephalopathy, chromosome abnormality, and congenital craniocerebral malformation. The Ethics Committee of the First Hospital of Jilin University approved the study (reference number 2020-312). All the patients were treated in accordance with the AAP guidelines for neonatal hyperbilirubinemia (10).

According to the case records of the children within 24 h of admission, the mental state, muscle tone, and crying of the children were evaluated by bilirubin-induced neurologic dysfunction protocol (BIND score) (13). The total BIND score of 1 to 3 indicates subtle, usually reversible, toxicity of bilirubin; scores of 4 to 6 are considered to reflect moderate but potentially reversible ABE; and scores of 7 to 9 suggest severe ABE, which may lead to long-term disability from kernicterus (13). In our study, ABE was defined as BIND ≥ 4.

### Data Collection

The demographic characteristics included GA, premature rupture of membranes, type of delivery, birth weight (BW), type of feeding, mother's age, hypertensive disorders in pregnancy, gestational diabetes mellitus, intrauterine distress, and meconium-stained amniotic fluid. The pathological information included cranial hematoma, intracranial hemorrhage, isoimmune hemolysis, sepsis, polycythemia, and asphyxia. The admission examination results included WBC, red blood cell (RBC), hemoglobin (Hb), platelet, TSB, C-reactive protein, albumin, blood glucose, and magnetic resonance imaging (MRI). Isoimmune hemolysis was defined as hemolysis confirmed by screening test, including ABO hemolysis, Rh hemolysis, or other hemolysis caused by maternal and infant blood group incompatibility. Sepsis was defined as a case of a newborn with clinical symptoms of sepsis and positive blood culture results. Polycythemia was defined as hematocrit >0.63. Neonatal asphyxia is defined as having the following four characteristics: (1) the umbilical artery demonstrates severe metabolic acidosis (pH < 7), (2) Apgar score <4, (3) neurological sequelae, and (4) multi-organ dysfunction. The normal values (14) of newborn BW, WBC, RBC, Hb, platelet, and blood glucose are defined in Table 1. MRI was performed with neonates in the sedated state using a 1.5-T whole-body MR scanner (Avanto, Siemens) with a standard circular polarized head coil. The conventional MRI scan sequences and parameters were as follows: a transverse T2-weighted turbo spin echo sequence (TR, 4,500 ms; TE, 82 ms) and sagittal and transverse T1-weighted sequences (TR, 415 ms; TE, 8.4 ms) with a slice thickness of 5 mm, a matrix of 205 × 256 pixels, and a field of view of 230 × 230 mm. Abnormal T1WI hyperintensity in the globus pallidus (GP) was judged by two experienced neuroradiologists by comparing it to the putamen of the same patient, and reference was made to the difference in GP and putamen T1WI intensity of normal infants.

**Table 1:** The normal values of newborn birth weight (BW), white blood cell (WBC), red blood cell (RBC), hemoglobin (Hb), platelet, and blood glucose.

**BW (kg)**	**WBC**	**RBC**	**Hb**	**Platelet**	**Blood glucose**
	(*10^**9**^/L)	(*10^**12**^/L)	**(g/L)**	(*10^**9**^/L)	**(mmol/L)**
2.5–4.0	5–21	2.8–5.2	140–200	100–300	2.8–5

### Statistical Analysis

Continuous variables were expressed as median (quartile range) and compared using Mann–Whitney test. Categorical variables were expressed as numbers (frequency) and compared using χ^2^ test. All variables significantly associated with ABE were used as candidates for multivariate logistic regression. A nomogram was developed based on the results of multivariate logistic regression analysis using the rms package of R, version 3.6.1. The nomogram is based on proportionally converting each regression coefficient in multivariate logistic regression into 0–100 points. The variable with the highest standardized β coefficient was assigned 100 points. The scores of each variable can be added to derive the total score, which corresponds to the prediction probability. The predictive performance of the nomogram was measured by concordance index (*C* index) and calibrated with 1,000 bootstrap samples to reduce the overfit bias. The optimal cutoff value determined by the maximum Youden index (sensitivity + specificity – 1) was calculated by receiver operating characteristic curve analysis. All analyses were performed using SPSS, version 23.0, and R, version 3.6.1. A *p*-value <0.05 (two-tailed) was considered to be statistically significant.

## Results

### Clinical Characteristics of the Subjects

A total of 517 newborns were included in this study, of which 102 (19.7%) had acute bilirubin encephalopathy. The clinical characteristics of the subjects with ABE and non-ABE were shown in Table 2. Significant differences were found in GA, feeding, mother's age, WBC, Hb, T1WI hyperintensity, TSB, and albumin between ABE and non-ABE patients.

**Table 2:** Clinical characteristics of the subjects.

	**Non-neonatal acute bilirubin encephalopathy (ABE) (*n* = 415)**	**ABE (*n* = 102)**	**χ^**2**^/Z**	***P***
Sex			0.587	0.443
Male	239 (57%)	63 (62%)		
Female	176 (43%)	39 (38%)		
Gestational age			5.814	0.016
≥37 weeks	364 (88%)	80 (78%)		
<37 weeks	51 (12%)	22 (22%)		
Premature rupture of membranes			0.086	0.769
No	295 (71%)	70 (70%)		
Yes	120 (29%)	30 (30%)		
Type of delivery			2.956	0.086
Spontaneous labor	308 (74%)	84 (83%)		
Cesarean delivery	107 (26%)	18 (18%)		
Birth weight			1.938	0.164
Normal	367 (88%)	85 (83%)		
Abnormal	48 (12%)	17 (27%)		
Type of feeding			5.609	0.018
Breast milk	166 (40%)	54 (53%)		
Others	249 (60%)	48 (47%)		
Mother's age			17.882	<0.001
≤35 years	369 (89%)	74 (73%)		
>35 years	46 (11%)	28 (27%)		
Hypertensive disorders in pregnancy			2.519	0.112
No	385 (93%)	99 (97%)		
Yes	30 (7%)	3 (3%)		
Gestational diabetes mellitus			1.160	0.282
No	392 (95%)	99 (97%)		
Yes	23 (5%)	3 (3%)		
Intrauterine distress			2.622	0.105
No	400 (96%)	102 (100%)		
Yes	15 (3%)	0 (0)		
Meconium-stained amniotic fluid			0.377	0.539
No	393 (95%)	95 (93%)		
Yes	22 (5%)	7 (7%)		
Intracranial hemorrhage			0.750	0.386
No	245 (59%)	65 (64%)		
Yes	170 (41%)	37 (36%)		
Cranial hematoma			0.719	0.396
No	313 (75%)	81 (79%)		
Yes	102 (25%)	21 (21%)		
Isoimmune hemolysis			0.272	0.602
No	286 (69%)	73 (72%)		
Yes	129 (31%)	29 (28 %)		
Sepsis			0.403	0.526
No	399 (96%)	96 (94%)		
Yes	16 (4%)	6 (6%)		
Polycythemia			2.450	0.118
No	408 (98%)	97 (95%)		
Yes	7 (2%)	5 (5%)		
Asphyxia			<0.001	1.000
No	409 (99%)	100 (98%)		
Yes	9 (1%)	2 (2%)		
White blood cell			37.192	<0.001
Normal	397 (96%)	79 (77%)		
Abnormal	18 (4%)	23 (23%)		
Red blood cell			1.627	0.202
Normal	303 (73%)	68 (67%)		
Abnormal	112 (27%)	34 (33%)		
Hemoglobin			16.125	<0.001
Normal	308 (74%)	55 (54%)		
Abnormal	107 (26%)	47 (46%)		
Platelet			3.506	0.061
Normal	234 (56%)	47 (46%)		
Abnormal	181 (44%)	55 (54%)		
Blood glucose			0.970	0.325
Normal	185 (45%)	51 (50%)		
Abnormal	230 (55%)	51 (50%)		
T1WI hyperintensity			143.698	<0.001
No	375 (90%)	38 (37%)		
Yes	40 (10%)	64 (63%)		
Total bilirubin, mg/dl	27.5 (26.2–29.9)	35.4 (29.9–39.5)	10.903	<0.001
γ-gt, U/L	126.9 (87.5–191.0)	139.0 (94.0–205.0)	0.995	0.320
C-reactive protein, mg/L	1.38 (0.50–4.33)	2.05 (0.50–6.10)	0.570	0.569
Albumin, g/L	35.8 (33.7–37.7)	35.1 (32.5–37.3)	2.289	0.022

### Multivariate Logistic Regression Analysis of ABE Presence

On multivariate analysis, with results reported as odds ratio (95% CI), T1WI hyperintensity [18.819 (8.838–40.069)], mother's age > 35 years [2.618 (1.096–6.2530)], and abnormal WBC level, TSB level [1.340 (1.242–1.445)], and albumin level [0.812 (0.726–0.907)] were independently associated with ABE (Table 3).

**Table 3:** Multivariate logistic regression analysis of neonatal acute bilirubin encephalopathy presence.

	**β**	**OR 95% CI**	***P***
T1W1 hyperintensity, yes vs. no	2.935	18.819(8.838–40.069)	<0.001
Mother's age, >35 vs. ≤ 35 year	0.963	2.618(1.096–6.253)	0.030
White blood cell, abnormal vs. normal	1.872	6.503(2.226–18.994)	0.001
Total bilirubin, mg/dl	0.292	1.340(1.242–1.445)	<0.001
Albumin, g/L	0.209	0.812(0.726–0.907)	<0.001

### Development and Validation of an ABE-Predicting Nomogram

**Figure 1:**
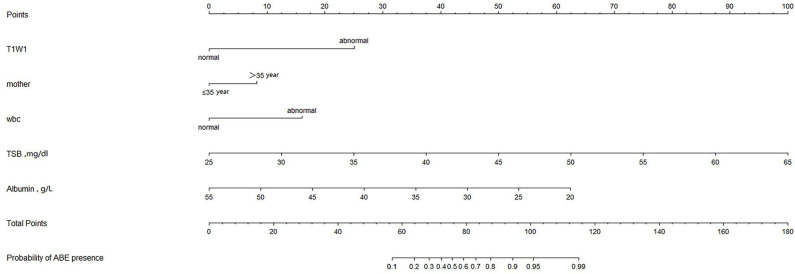
Nomogram of ABE Presence.

**Figure 2:**
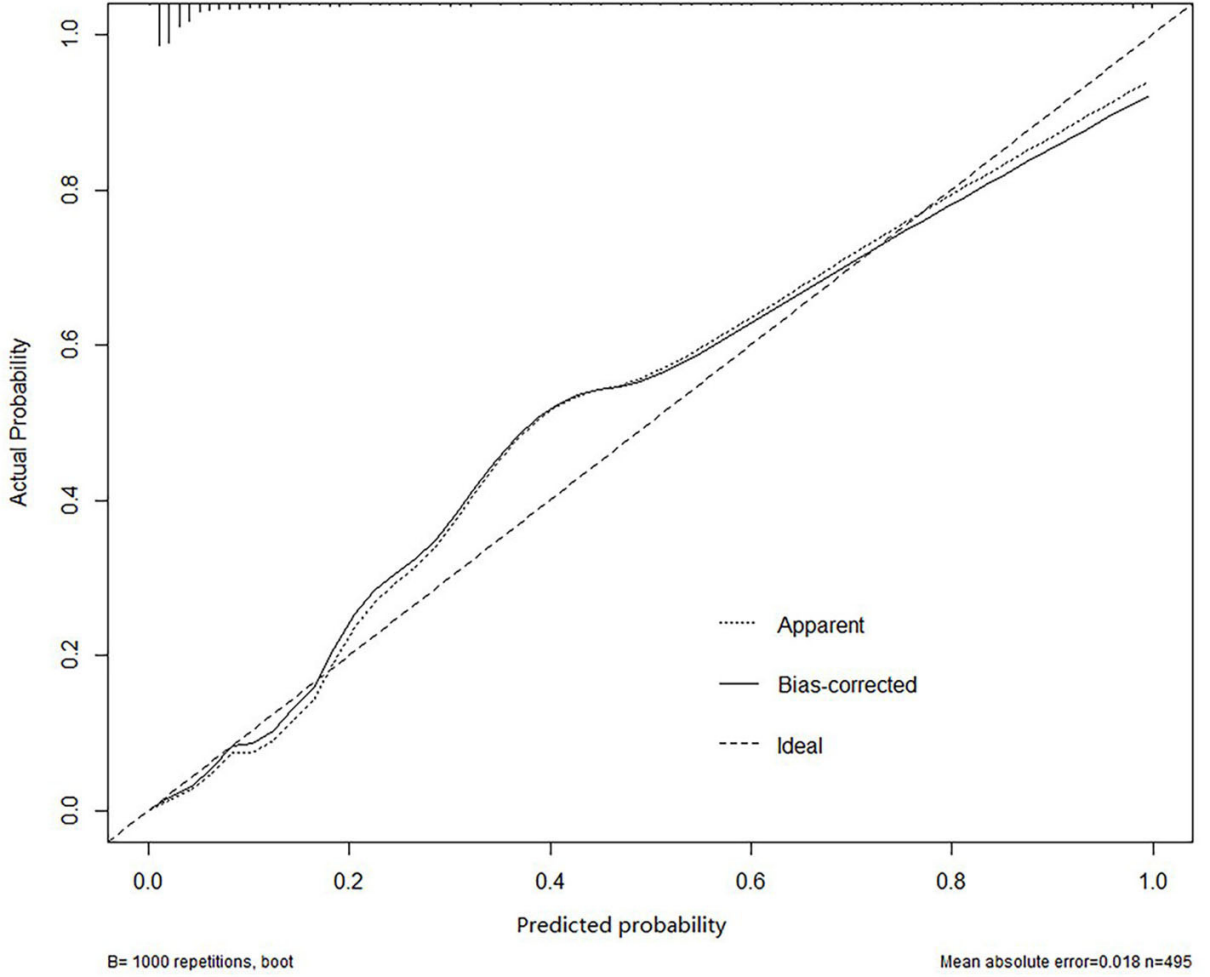
The result of bootstrap validation.

All independently associated risk factors were used to form an ABE risk estimation nomogram (Figure 1). The bootstrap validation method was used to internally validate the resulting model. The nomogram demonstrated good accuracy in predicting the risk of ABE, with an unadjusted *C* index of 0.943 (95% CI, 0.919–0.962) and a bootstrap-corrected *C* index of 0.900 (Figure 2). The optimal cutoff value of the total nomogram scores was determined to be 62.8. The sensitivity, specificity, positive predictive value, and negative predictive value when used in differentiating the presence from the absence of ABE were 88.9, 89.9, 66.1, and 97.3%, respectively.

## Discussion

Our study suggests that T1 signal enhancement, mother's age > 35 years, and abnormal WBC, TSB, and albumin levels were significantly associated with ABE. Previous studies (6, 15–17) have attempted to use blood biomarkers and imaging to predict ABE. However, the predictive effect was considered to be unsatisfactory (11, 12). A recent study reported a predictive model that included three factors (TSB, bilirubin/albumin ratio, and sepsis) in the risk assessment of ABE (18), but further clinical validation is needed. Furthermore, the scores obtained by this prediction model cannot be directly converted into the prediction probability, which may cause confusion for users. Among the currently available prediction tools, a nomogram can provide an individualized, highly accurate risk assessment and is easy to use (19). To our knowledge, we have established the first ABE risk assessment, which includes five easily available variables. The good performance of this model is supported by the *C* index value of 0.943, and the optimal calibration curve shows a good consistency between the prediction and the actual observation.

TSB is a recognized predictor of bilirubin neurotoxicity. Studies have shown that ABE, neurological deafness, or jaundice may occur in newborns with TSB ≥ 20 mg/dl or higher (20–23). Bhutani et al. (24) reported that the risk of ABE was 6% when TSB ≥ 25 mg/dl and 14–25% when TSB ≥ 30 mg/dl, and ABE was present in almost all children with TSB ≥ 35 mg/dl. It is worth noting that the TSB level of newborns often fluctuates, and some of them reach an extremely high level of TSB without neurological damage (12). To reduce overtreatment, TSB should be combined with other indicators in ABE's prediction.

Albumin usually appears as bilirubin/albumin ratio in ABE's predictions. The bilirubin/albumin ratio is used as a surrogate for plasma-free bilirubin because it contains two of the three factors (TSB, albumin, and albumin binding affinity) that determine plasma-free bilirubin (25). However, whether bilirubin/albumin ratio can improve the prediction over TSB alone remains controversial (17, 18). We think that it is more reasonable to analyze the effect of albumin on ABE in the prediction model already included in TSB. In addition, albumin values are more available than bilirubin/albumin ratio in clinical applications.

MRI is increasingly used to image the neonatal brain affected by bilirubin toxicity. Studies have shown that MRI may show T1WI hyperintensity in ABE neonates (26, 27). Normal myelination in newborns of the same age can also lead to this change in the same regions similar to the bilirubin-induced ABE (15). Therefore, T1WI hyperintensity cannot be used as a specific diagnostic indicator for ABE. Our study shows that T1WI hyperintensity can be combined with other indicators to predict ABE.

An abnormal level of WBC is considered to be a cause of jaundice (28), and our results suggest that it is also a risk factor for ABE. The abnormal level of WBC is mostly associated with infection, which can lead to the destruction of red blood cells, decreased uptake of binding bilirubin, inhibition of rate-limiting enzyme activity in the liver, and increase in the severity of jaundice. The increased blood–brain barrier permeability caused by a concurrent infection also further aggravates the neurotoxic effects of bilirubin (13). Zhang et al. (18) reported that sepsis was a risk factor for ABE, but there was no significant difference between ABE and non-ABE neonates in this study. This may be related to the low positive rate of blood culture. In the previous study, sepsis was defined as an abnormal level of WBC, positive C-reactive protein, or positive blood cultures. In this study, sepsis was defined as a newborn with both clinical symptoms of sepsis and positive blood culture results. Clinically, sepsis is not a good predictor of ABE because it takes a long time to be confirmed by blood culture. Our results suggest that the true risk factor of ABE may be the abnormal level of WBC rather than sepsis.

Although there was no report on the association between ABE presence and Mother's age, being an older pregnant woman is considered a risk factor for a variety of neonatal diseases, such as hemolytic diseases, prematurity, asphyxia, and infection, which may contribute to the presence of ABE (10, 29). Our results showed that some influencing factors of hyperbilirubinemia, such as polycythemia, asphyxia, and cranial hematoma, had no effect on ABE. This may be due to the fact that our study population included newborns with EHB and mother's age was included as a variable.

In the nomogram, “points” represented the scores corresponding to each risk factor, and “total points” was obtained by adding the five risk factor scores for each newborn. According to the “total points” of each newborn, the probability of ABE presence can be obtained. We summarized the sensitivity, specificity, positive predictive value, and negative predictive value using 62.8 as the cutoff value to evaluate the clinical application of the model. Based on these predictions, nomograms can be used as a tool to guide the treatment of neonates with hyperbilirubinemia and reduce the incidence of ABE. There are some limitations in our study: (1) The result is based on data from a single institution and needs to be validated by data from other centers; (2) this study is retrospective and more prospective studies are needed to verify the reliability of the model; and (3) due to the limitation of sample size, this study only conducted internal verification, and more data were needed for external verification.

## Conclusions

A nomogram was constructed using five risk factors of ABE. This model can help clinicians to determine the best treatment for neonatal hyperbilirubinemia.

## Data Availability Statement

The raw data supporting the conclusions of this article will be made available by the authors, without undue reservation.

## Ethics Statement

The studies involving human participants were reviewed and approved by The Ethics Committee of the first hospital of Jilin University approved the study (Reference Number: 2020-312). Written informed consent to participate in this study was provided by the participants' legal guardian/next of kin.

## Author Contributions

HW and YQ conceptualized and designed the study, drafted the initial manuscript, and reviewed and revised the manuscript. SH designed the data collection instruments, collected data, carried out the initial analyses, and reviewed and revised the manuscript. XF conceptualized and designed the study, coordinated and supervised the data collection, and critically reviewed the manuscript for important intellectual content. All authors approved the final manuscript as submitted and agree to be accountable for all aspects of the work.

## Conflict of Interest

The authors declare that the research was conducted in the absence of any commercial or financial relationships that could be construed as a potential conflict of interest.

## Abbreviations

ABE: neonatal acute bilirubin encephalopathy
EHB: extreme hyperbilirubinemia
TSB: total bilirubin
BIND score: bilirubin-induced neurologic dysfunction protocol
GA: gestational age
PROM: premature rupture of membranes
BW: birth weight
HDIP: hypertensive disorders in pregnancy
GDM: gestational diabetes mellitus
MSAF: meconium-stained amniotic fluid
Hb: hemoglobin.
